# Items

**Published:** 1879-03

**Authors:** 


### Items.

BULLETIN OF THE PUBLIC HEALTH, Issued by the Surgeon-General U. S. Marine Hospital Service under the National Quarantine Act of 1878. Week ended February 15th, 1879. No. 32.

OFFICE SURGEON-GENERAL M. H. S.
WASHINGTON, Feb. 19th, 1879.

*Boston.*—Week ended Feb. 15th. Deaths from all causes, 155, an annual ratio of 22.1 per 1,000 of the population; 18 cases of scarlet fever, 2 deaths; 22 cases of diphtheria, 9 deaths. Bronchitis caused 6 deaths, pneumonia 19, phthisis 26.

*Providence.*—Week ended Feb. 15th. Total deaths, 41. An. ratio, 21; 5 deaths from scarlet fever, 5 from diphtheria. "Scarlet fever prevalent and increasing."

*New York.*—Week ended Feb. 15th. Total deaths, 544. An. ratio, 26.3; 69 deaths from scarlet fever; 12 from diphtheria, 18 from whooping-cough, 35 from bronchitis, 55 from pneumonia, 101 from phthisis.

*Hudson County N. J.* (Including Hoboken and Jersey City.)—Week ended Feb. 15th. Total deaths, 61. An. ratio, 16. Enteric fever caused 3 deaths, scarlet fever 2, diphtheria 3, acute lung diseases 9, phthisis 10.

*Brooklyn.*—Week ended Feb. 15th. Total deaths, 222. An. ratio, 20.4; 5 cases of enteric fever, 2 deaths; 71 cases of scarlet fever, 9 deaths; 44 cases of diphtheria, 11 deaths; 40 deaths from acute respiratory diseases, 33 from phthisis.

*Buffalo.*—Week ended Feb. 15th. Total deaths, 31. An. ratio, 12; 4 deaths from scarlet fever, 2 from diphtheria.

*Philadelphia.*—Week ended Feb. 15th. Total deaths, 349. An. ratio, 21. Enteric fever caused 5 deaths, scarlet fever 9, diphtheria 9, acute pulmonary diseases 71, phthisis 60.

*Pittsburgh.*—2 weeks ended Feb. 15th. Total deaths 121. An. ratio, 21.7. Enteric fever caused 5 deaths, scarlet fever 3, diphtheria 11 deaths.

*Baltimore.*—Week ended Feb. 15th. Total deaths, 160. An. ratio, 22.8 ; 1 death from enteric fever, 6 from scarlet fever, 10 from diphtheria, 31 from acute pulmonary diseases, 25 from phthisis.

*District of Columbia* (Including Washington and Georgetown). Week ended Feb. 15th. Total deaths, 87. An. ratio, 28.3. Scarlet fever caused 3 deaths, diphtheria 2, acute pulmonary diseases 18, phthisis 18.

*Chicago.*—2 weeks ended Feb. 15th. Total deaths, 244. An. ratio, 14. Enteric fever caused 6 deaths, scarlet fever 5, diphtheria 16, acute lung diseases 42.

*Milwaukee.*—Week ended Feb. 15th. Total deaths, 40 ; rate, 18 ; 5 deaths from diphtheria.

*Louisville.*—Week ended Feb. 15th. Total deaths, 58. Annual ratio, 19. Diphtheria caused 1 death, acute lung affections 16, phthisis 8.

*St. Louis.*—2 weeks ended Feb. 15th. Total deaths, 215. An. ratio, 11.2. Enteric fever caused 1 death, scarlet fever 2, diphtheria 5, phthisis 36, pneumonia 29.

*Richmond.*—2 weeks ended Feb. 15th. Total deaths, 67. An. ratio, 22. Scarlet fever caused 8 deaths, acute lung diseases 9, phthisis 8.

*Savannah.*—2 weeks ended Feb. 15th. Total deaths, 41. An. ratio, 38. Death-rate among the negroes twice as great as among the whites.

*Mobile.*—Week ended Feb. 15th, 17 deaths. An. ratio, 22; 1 death from diphtheria. "City very healthy with exception of epidemic of influenza."

*New Orleans.*—Week ended Feb. 9th. Total deaths 87. An. ratio, 22. Acute lung diseases caused 14 deaths, phthisis 17. No deaths reported from scarlet fever or diphtheria.

*San Francisco.*—Week ended Feb. 7th. Total deaths, 101.

An. ratio, 17.5. Enteric fever caused 2 deaths, scarlet fever 1, diphtheria 4, acute lung diseases 18, phthisis 15.

*Havana.*—Week ended Feb. 15th. Yellow fever caused 1 death, small-pox 15.

*Great Britain.*—Week ended Feb. 1st. The average death-rate of the 23 large cities was 28. Dublin, 49; Liverpool, 36; Manchester, 35; Glasgow, 29; Edinburgh, 25; Sheffield, 25.

*London.*—1821 deaths. An. ratio, 26. Measles caused 23 deaths, scarlet fever 33, diphtheria 14, whooping-cough 64, acute lung diseases 541; 28 deaths from small-pox in London; 21 in Dublin.

*German Empire.*—2 weeks ended Jan. 18th. In 150 cities there were 7,446 deaths, an average annual rate of 26 per 1,000. Measles caused 94 deaths, scarlet fever 184, diphtheria 352, whooping-cough 105, acute lung diseases 666, phthisis 1,015.

*Paris.*—Jan. 24th to 30th. Deaths, 1,109. Small-pox caused 8, diphtheria 28, enteric fever 21.

*Vienna.*—Jan. 19th to 25th. Deaths, 42. Rate, 30. Small-pox caused 13 deaths, diphtheria 21.

*St. Petersburgh.*—Jan. 12th to 18th. Deaths, 623. Rate, 48. Small-pox caused 58 deaths.

*Rio de Janeiro.*—2 weeks ended Jan. 18th. Deaths, 393. An. ratio, 34. Yellow fever caused 30 deaths, small-pox 30, enteric fever 11, "pernicious" fever 20. No deaths from diphtheria or scarlet fever reported.

The U. S. Consul at Pernambuco reports that in the interior of the province of Ceara a severe drought has prevailed for two years and a half, no rain having fallen during that time. The excessive dryness caused the disappearance of the innumerable small streams which furnished the whole water supply of the country, the consequent death of nearly all the cattle and sheep, and the complete destruction of the usual means of subsistence of the population, which is wholly an agricultural one. The people have been reduced to subsistence on roots, cotton-pods, reptiles and any living or dead thing that would sustain life, some resorting even to cannibalism. In the winter of 1878 small-pox appeared in epidemic form and caused a frightful mortality among the starving people. A general flight of the people from the interior to the coast cities occurred. The normal population of 25,000 in Fortaleza, the capital, was quickly raised to 100,000, the squares of the city being filled with thousands of unsheltered people dying of disease and starvation. One-half of the original population of the city have died of small-pox. In the new cemetery of Lagoa Funda, opened in the middle of last year, there were 60,000 interments up to Jan. 1st. The number of burials from small-pox alone between Nov. 1st and Jan. 1st in this cemetery were 24,470; the total interments in the city for the two months being 31,571. At Parahyba 12,000 refugees out of 15,000 who had fled to the port died, and similar distressing accounts are given of the other coast cities. The Consul estimates the usual population at 900,000, of whom 500,000 have died of disease and starvation. The Brazilian Government have expended $10,000,000 for the relief of the sufferers. At last advices slight rains have fallen in the interior, and it is believed that the worst period of the scourge has been passed.

JNO. M. WOODWORTH,
Surgeon-General, U. S. Marine Hospital Service.

AN ATTEMPT AT BLACKMAILING A PHYSICIAN, in which the other party got the worst of it.

In August of last year, a certain Joseph Clem received an injury in his right eye from being struck by a gun cap; he consulted Dr. M. F. Coomes, of Louisville, and put himself under his care for some time. He did not pay the doctor's bill for the sum of twenty-two dollars, but sued him for malpractice, alleging "that his eye was unskillfully treated by the defendant, while treating his eye as his physician and oculist, and that, by reason of said unskillful treatment, he has been damaged to the extent of one thousand dollars ($1.000), and that he fears he will lose entirely the sight of said eye because of said unskillful treatment."

Dr. Coomes, in his answer, makes the following statement: "The lower margin of the right eye was wounded about a quarter of an inch from the inner angle; that there was a rent in the conjunctiva about one-third of an inch in length, running downward and inward from the margin of the cornea, and there was also a corresponding rent in the sclera, which extended on into the cornea for a line or a line and a half.

There was a considerable rent in the inner and inferior quadrant of the iris, and a portion of the iris was between the lips of the wound, and was dragged out until it was almost in contact with the margin of the lower lid. The pupil was elongated downward and inward, the wound extending through the ciliary region, with considerable hemorrhage from the wound.

The plaintiff at this time could distinguish light from darkness. He said the light looked reddish, and he could not give the definite outline of any object. The cavity of the globe was filled with blood, so as to render an ophthalmoscopic examination impossible. The iris was almost hidden by blood, and the pupil was completely closed with it."

A solution of atropia (0.25 to 30.0), was dropped into the eye, and a wet compress applied.

"Defendant says that when he looked at the eye he told the plaintiff that the chances were against its recovery for visual purposes; that if any portion of the cap was buried within the cavity of the globe, the eye would be lost for visual purposes, and that even if the foreign substance was not there it was not likely the eye would ever be entirely recovered; but every chance was given the eye of the plaintiff to allow the hæmorrhage to clear up and permit a more thorough examination. Defendant told the plaintiff at the time that if the foreign body was in the cavity of the globe, violent inflammation would intervene, and that it would then be safe to remove the globe at once—inflammation did afterwards set in.

Defendant requested plaintiff to consult any physician or oculist he desired, and assured him the exercise of such a right would cause him (the defendant) no unkindly feeling, but, on the contrary, he would be gratified if the opinion of some one else would be had.

Plaintiff did go to another oculist, and made an appointment to have the injured eye removed. But for some reason unknown to defendant, this was not done."

Before the doctor's answer was presented to the court, the attorney for the plaintiff attempted to compromise the suit.

These attempts were renewed from time to time, first offering to accept $500, then $100, then $50; and, finally, after failing to induce the court to strike out all that part of the defendant's answer relating to the treatment of the plaintiff, his attorney took a copy of the answer and went around the city trying to find some doctor willing to express an unfavorable opinion of defendant's treatment of plaintiff. Failing in this, he refuses to go to trial, and moves the dismissal of the suit he has brought.

Whereupon the court dismissed the suit, but, at the same time adjudged that the defendant recover of the plaintiff his costs herein expended, and may have execution.

---

TESTS FOR CARBOLIC AND CRESYLIC ACIDS, AND CREASOTE. (*Chemist and Druggist.*)

Pure carbolic acid takes up 26 to 27 per cent. of water to form a hydrous acid. Cresylic acid absorbs only 13 or 14 per cent. Absolute carbolic acid is solid at ordinary temperatures, and the hydrous acid in freezing mixtures. Neither absolute nor hydrous cresylic acid, nor creasote show any signs of freezing on exposure to the same cold. Absolute carbolic acid dissolves in 10.7 parts of cold water; absolute cresylic acid in 31 parts. Benzol mixes with either in any proportions and separates the water from the hydrous acids; benzoline mixes with cresylic acid and creasote in all proportions, but carbolic acid will not take up more than half its volume. The two acids mix in any proportion with Price's glycerin, but creasote will not dissolve in it. A mixture of one part of carbolic acid with one of glycerin is not precipitated by the addition of three parts of water; a similar mixture of cresylic acid and glycerin is precipitated by adding two parts of water. The addition of one drop of a 10 per cent. aqueous solution of ferric chloride to 15 C. C. of an aqueous solution of either acid causes a permanent violet-blue coloration; when creasote is similarly tested a blue color results, but almost instantly changes to green or brownish yellow.

Cresylic acid constitutes an ingredient or impurity of commercial carbolic acid.

